# Operative Strategies for Aplastic Circle of Willis Arteries on CTA in Meningioma Surgery: A Case Report

**DOI:** 10.1227/neuprac.0000000000000058

**Published:** 2023-10-09

**Authors:** Joanna K. Tabor, Alexandros F. Pappajohn, Haoyi Lei, Joseph O'Brien, Robert K. Fulbright, Saul F. Morales-Valero, Jennifer Moliterno

**Affiliations:** * Department of Neurosurgery, Yale School of Medicine, New Haven, Connecticut, USA;; ‡ The Chênevert Family Brain Tumor Center, Smilow Cancer Hospital, New Haven, Connecticut, USA;; § Department of Radiology and Biomedical Imaging, Yale School of Medicine, New Haven, Connecticut, USA

**Keywords:** Sphenoid wing meningioma, Anterior cerebral artery, Vascular encasement, Resection, Case report, Aplastic

## Abstract

**BACKGROUND AND IMPORTANCE:**

Meningiomas frequently involve critical neurovascular structures. Preoperative imaging with computed tomography angiography (CTA) can help understand the relationship of tumor with neurovascular structures. Although CTA was useful in preoperative planning and less invasive, we present a unique case in which it mistakenly represents a hypoplastic anterior cerebral artery as aplastic and thus displays poor sensitivity in the Circle of Willis.

**CLINICAL PRESENTATION:**

A 66-year-old woman presented with new onset seizures and MRI demonstrated a sphenoid wing meningioma with tumor involvement of the internal carotid artery and right M1 artery. On preoperative CTA, a right A1 artery was not appreciated. However, a hypoplastic right A1 artery was identified during careful dissection guided by micro-Doppler and found to be encased within the tumor. All arteries were preserved, and a near-total resection was achieved with a small remnant of tumor at the supraclinoid internal carotid artery.

**CONCLUSION:**

Although CTAs can be useful in understanding vascular anatomy and its association with tumors, they are not ideal for providing information about hypoplastic arterial segments. Seemingly aplastic arteries on CTA may very well be present, albeit hypoplastic. While we recognize the usefulness of a less invasive modality of CTA to help guide surgical strategy, we underscore recognizing this potential pitfall and recommend the use of the microvascular Doppler during careful dissection when working within tumor in the location of a seemingly aplastic artery.

**ABBREVIATIONS:**
COW: circle of Willis
FL: frontal lobe
ON: optic nerve
SWM: sphenoid wing meningioma
TL: temporal lobe.

Skull base meningiomas can often involve critical neurovascular structures. Sphenoid wing meningiomas (SWMs) that are large and medially located can present a particular operative challenge as they often encase the internal carotid artery (ICA) and its terminus, bifurcation and the middle cerebral artery, anterior cerebral artery, and optic nerves.^1^ Extent of resection is associated with meningioma recurrence, and the degree of vascular encasement and cavernous sinus involvement can affect the extent of resection.^2^ Concomitant circumferential encasement of the supraclinoid ICA, M1, and A1 artery is associated with an increased risk for stroke, and thus, subtotal resection is often necessary to limit potential postoperative complications.^2^

Imaging to identify the degree of involvement of critical vasculature, such as computed tomography angiography (CTA) or magnetic resonance angiography, is useful for preoperative planning. CTA is favored over conventional angiogram as it is noninvasive and able to assess arteries as small as 1 mm in diameter and provide 3-dimensional visualization of vasculature.^3^ However, CTA has a tendency to underestimate arterial segments and demonstrates poor sensitivity in detecting hypoplastic arteries as compared with more invasive digital subtraction angiography (DSA).^4^ In this case, we present a 66-year-old woman with a SWM encasing the ICA and right M1 artery. Although the right A1 artery was noted to be absent on routine preoperative CTA, a hypoplastic right A1 was identified intraoperatively with careful microdissection guided by the micro-Doppler. This case underscores the importance of not relying solely on preoperative vascular imaging and assuming that important arteries are absent when they may be hypoplastic and functional within the tumor.

## CLINICAL PRESENTATION

**FIGURE 1.**
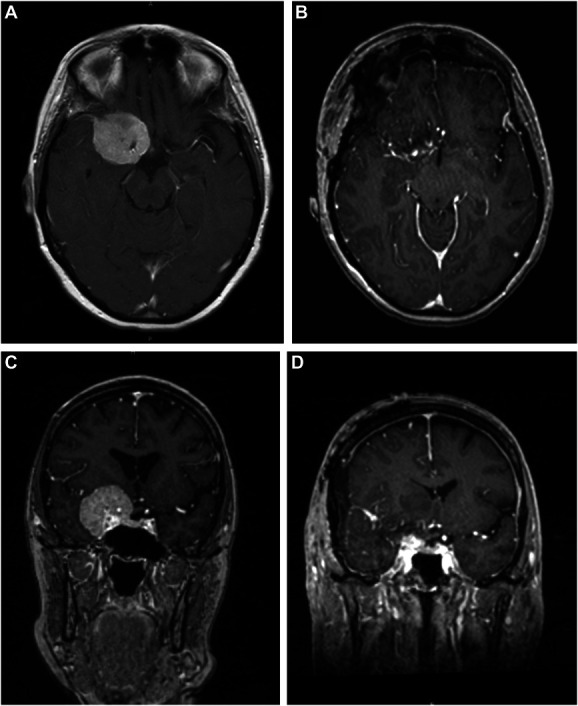
Axial view of T1-weighted MRI with contrast demonstrating a right sphenoid wing meningioma **A**, before and **B**, after resection. Coronal view of T1-weighted MRI **C**, before and **D**, after resection demonstrating a near-total resection, with small residual tumor encasing the right supraclinoid internal carotid artery at its intracranial origin.

A 66-year-old right-handed woman presented with new-onset seizure. Her neurological examination was unremarkable, and she was started on lamotrigine at an outside hospital. MRI of the brain demonstrated a 4-cm dural-based mass extending along the right sphenoid wing, with extension along the anterior clinoid process and into the cavernous sinus and suprasellar cistern (Figure 1). There was mass effect on the adjacent frontal and temporal lobes with associated edema.

**FIGURE 2.**
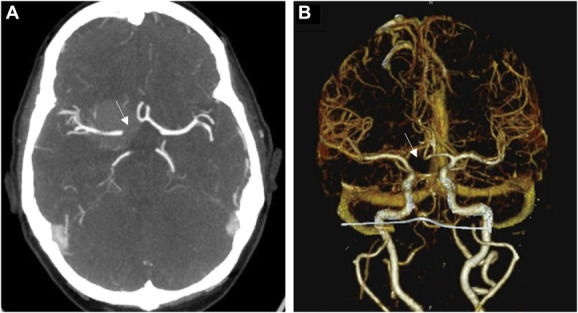
**A**, Axial view of CTA and **B**, 3-dimensional reconstruction of CTA demonstrating missing right A1 artery (arrow). CTA, computed tomography angiography.

**FIGURE 3.**
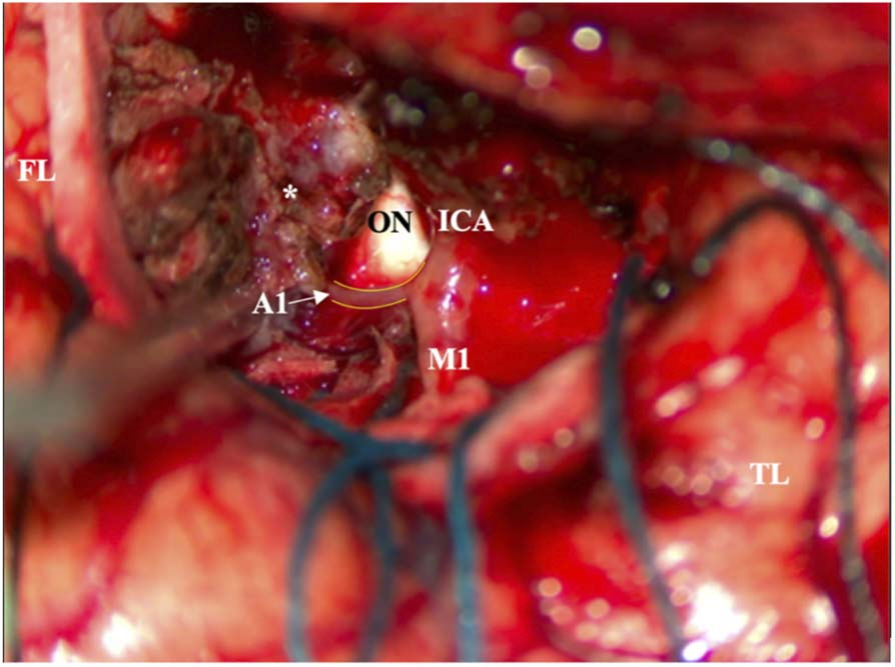
Intraoperative photograph demonstrating the ICA, M1 artery, and patent, but hypoplastic, A1 artery (arrow) encased by the tumor (asterisk). The ON is visualized as well as the FL and TL. FL, frontal lobe; ICA, internal carotid artery; ON, optic nerve; TL, temporal lobe.

As standard for the senior author, a preoperative CTA was obtained and demonstrated encasement of the supraclinoid portion of the right ICA, its bifurcation, and most of the M1. Notably, no right A1 artery was appreciated and was believed likely to be aplastic (Figure 2). A lumbar drain was placed preoperatively to aid with retractor-less surgical resection.^5^ Informed consent was obtained, and a modified pterional craniotomy was performed with intraoperative neuromonitoring, including motor evoked potentials, somatosensory evoked potentials, and electroencephalogram. Extradural drilling of the anterior clinoid process was performed which provided excellent access and exposure. Tumor blood supply was eradicated at the skull base under loupe magnification, and 30 cc of cerebrospinal fluid was released for brain relaxation. During routine microsurgical resection of the tumor, the micro-Doppler was used for the identification of arteries known to be embedded within the tumor and in anticipation of potential hypoplastic artery. A distal middle cerebral artery branch was identified and followed proximally, with further tumor debulking, leading to the ICA bifurcation. Here, an encased, but patent, hypoplastic right A1 artery was encountered (Figure 3) (Video). A near-total resection was achieved with a small remnant of tumor at the skull base at the origin of the supraclinoid ICA. There were no intraoperative complications. Histopathology demonstrated a World Health Organization Grade 1 meningioma with a low proliferative index, and whole-exome sequencing revealed a somatic driver mutation in *SMO* and no copy number variations. The patient had an uneventful postoperative course and follows with regular surveillance imaging with no reported changes in postoperative status.

## DISCUSSION

Despite the importance of neurovascular imaging for operative safety in SWM resection, there is a paucity of literature comparing the usefulness of different modalities in tumor surgery.^4^ Although DSA with catheter angiogram is the “gold standard” for the assessment of circle of Willis (COW) arterial anatomy, its invasiveness has rendered it a less favorable option. Meanwhile, CTA has shown to be an effective, less invasive alternative, particularly for aneurysm imaging and surgical planning.^4^ Although CTA and DSA have a concordance rate of 92.4% in elucidating COW arterial anatomy, CTA underestimates smaller arterial segments compared with DSA and has relatively poor sensitivity (52.6%) in identifying hypoplastic arteries.^4^ CTA detects normal-sized and aplastic segments with specificity and sensitivity greater than 90%, but its performance drops dramatically in detecting those that are hypoplastic.^4^ The caliber of vessel that CTA is able to detect varies across studies, with the minimum detectable diameter ranging from 0.6 to 1 mm.^3,6^

There is considerable structural variation in the COW throughout the population, with hypoplasia reported most often and present in 1%–13% of anterior cerebral artery segments. This, coupled with tumor-related stenosis and flow changes, should caution the operating neurosurgeon with extra vigilance when resecting medial SWMs in particular.^7^ Although the authors still support the use of CTA in understanding the vascular anatomy relative to the tumor, we advise that the neurosurgeon should remain cognizant that hypoplastic arterial segments may be present in areas of tumor encasement that seem aplastic on CTA. Therefore, we emphasize the necessity of understanding the potential pitfall of this otherwise useful preoperative planning adjunct and recommend extra attention and careful microsurgical dissection when working within tumor in the location of a seemingly aplastic artery.

Although caution when working along the COW is standard practice among skull base and vascular neurosurgeons, the identification of normal anatomy first and careful tracing of vascular structures is particularly crucial in these cases. Fine-tip bipolars, relatively smaller suction, and sharp dissection with micro-Doppler guidance can allow for a more controlled resection, as opposed to ultrasonic aspirators. The use of micro-Doppler in such cases is helpful as a surgical adjunct and has been shown to be a useful method for cerebral blood flow assessment in microsurgical intracranial aneurysm clipping.^8^ One should be cautious and check that the micro-Doppler is working properly first by assessing blood flow in normal, accessible blood vessels, before using it to guide tumor resection.

It is a standard practice of the senior author to commonly evaluate intracranial vasculature with CTA and to pursue DSA in cases in which more dynamic imaging could provide additional information and further guide surgical strategy. In the current case, DSA was not undertaken because it was the intent of the senior author to explore that particular area of tumor more cautiously with Doppler guidance and dissection to assess whether the artery was indeed aplastic or hypoplastic. This case, however, highlights the need for the understanding of this pitfall of CTA and that in the setting of a seemingly aplastic artery, DSA and/or more focused intraoperative attention is needed to avoid unintended injury and arterial sacrifice. Indeed the CTA and DSA are complimentary as DSA can be superior at identifying smaller caliber vessels and those near the skull base, although CTA is useful in offering a 3-dimensional relationship of the tumor with surrounding vasculature.^9^ The additional benefit of DSA in conjunction with CTA can be decided on a case-by-case basis and may vary neurosurgeon-by-neurosurgeon based on the justification of the need for a more invasive diagnostic procedure.

### Limitations

This case report indicates that the neurosurgeon should consider CTA as an informative, but not definitive, tool in elucidating COW arteries in planning tumor resection. However, it is limited in scope as it presents 1 patient. More cases would provide stronger evidence that CTA is only adjunct to the more important sound surgical judgment and careful COW arterial elucidation intraoperatively.

## CONCLUSION

Although CTAs can be useful in understanding vascular anatomy and its association with tumors, they are not ideal for providing information about hypoplastic arterial segments. Seemingly aplastic arteries on CTA may very well be present, albeit hypoplastic. Although we recognize the usefulness of the less invasive modality of CTA to help guide surgical strategy, we underscore recognizing this potential pitfall and recommend the use of the microvascular Doppler during careful dissection when working within tumor in the location of a seemingly aplastic artery.

## References

[1] Champagne PO Lemoine E Bojanowski MW. Surgical management of giant sphenoid wing meningiomas encasing major cerebral arteries. Neurosurg Focus. 2018;44(4):e12. 10.3171/2018.1.FOCUS17718 29606042

[2] McCracken DJ Higginbotham RA Boulter JH Degree of vascular encasement in sphenoid wing meningiomas predicts postoperative ischemic complications. Neurosurgery. 2017;80(6):957-966. 28327941 10.1093/neuros/nyw134

[3] Suzuki S Furui S Kaminaga T Yamauchi T. Measurement of vascular diameter in vitro by automated software for CT angiography: effects of inner diameter, density of contrast medium, and convolution kernel. Am J Roentgenol. 2004;182(5):1313-1317. 15100138 10.2214/ajr.182.5.1821313

[4] Han A Yoon DY Chang SK Accuracy of CT angiography in the assessment of the circle of Willis: comparison of volume-rendered images and digital subtraction angiography. Acta Radiol. 2011;52(8):889-893. 21828003 10.1258/ar.2011.110223

[5] Vetsa S Nadar A Vasandani S Criteria for cerebrospinal fluid diversion in retractorless sphenoid wing meningioma surgery: a technical report. J Neurol Surg Rep. 2022;83(03):e100-e104. 36060292 10.1055/s-0042-1753518 PMC9439877

[6] Skutta B Fürst G Eilers J Ferbert A Kuhn FP. Intracranial stenoocclusive disease: double-detector helical CT angiography versus digital subtraction angiography. AJNR Am J Neuroradiol. 1999;20(5):791-799. 10369348 PMC7056155

[7] Pentyala S Sankar KD Bhanu PS Kumar NSS. Magnetic resonance angiography of hypoplastic A1 segment of anterior cerebral artery at 3.0-Tesla in Andhra Pradesh population of India. Anat Cell Biol. 2019;52(1):43-47. 30984451 10.5115/acb.2019.52.1.43 PMC6449583

[8] Pereira BJ Holanda VM Giudicissi-Filho M Borba LA de Holanda CV de Oliveira JG. Assessment of cerebral blood flow with micro-Doppler vascular reduces the risk of ischemic stroke during the clipping of intracranial aneurysms. World Neurosurg. 2015;84(6):1747-1751. 26216705 10.1016/j.wneu.2015.07.042

[9] Han X Zhan Y Chen J. Comparative study of multi-slice CT angiography with digital subtraction angiography in the blood supply of meningiomas. Exp Ther Med. 2012;3(1):31-36. 22969840 10.3892/etm.2011.354 PMC3438678

